# Blood Biomarker Detection Using Integrated Microfluidics with Optical Label-Free Biosensor

**DOI:** 10.3390/s24206756

**Published:** 2024-10-21

**Authors:** Chiung-Hsi Li, Chen-Yuan Chang, Yan-Ru Chen, Cheng-Sheng Huang

**Affiliations:** Department of Mechanical Engineering, National Yang Ming Chiao Tung University, Hsinchu 30010, Taiwan; jyongsi0901.en10@nycu.edu.tw (C.-H.L.); zyzhang.en11@nycu.edu.tw (C.-Y.C.); yanru.en12@nycu.edu.tw (Y.-R.C.)

**Keywords:** optical biosensor, guided-mode resonance, lab-on-a-chip, optofluidics

## Abstract

In this study, we developed an optofluidic chip consisting of a guided-mode resonance (GMR) sensor incorporated into a microfluidic chip to achieve simultaneous blood plasma separation and label-free albumin detection. A sedimentation chamber is integrated into the microfluidic chip to achieve plasma separation through differences in density. After a blood sample is loaded into the optofluidic chip in two stages with controlled flow rates, the blood cells are kept in the sedimentation chamber, enabling only the plasma to reach the GMR sensor for albumin detection. This GMR sensor, fabricated using plastic replica molding, achieved a bulk sensitivity of 175.66 nm/RIU. With surface-bound antibodies, the GMR sensor exhibited a limit of detection of 0.16 μg/mL for recombinant albumin in buffer solution. Overall, our findings demonstrate the potential of our integrated chip for use in clinical samples for biomarker detection in point-of-care applications.

## 1. Introduction

Microfluidic technology, also known as lab-on-a-chip (LOC) technology, involves the integration of several functional devices or processes onto a single platform or chip. This technology offers the advantages of batch processing at a low cost and a low sample volume requirement, accelerating biochemical reactions in microscale or nanoscale channels and automating fluid handling processes [1]. Over the past decades, LOC technology has substantially evolved and now exhibits great promise for point-of-care (POC) applications in medical diagnostics [2,3].

Clinical tests of blood and urine samples play a key role in routine medical diagnostics. These tests often involve sample preprocessing and analyte measurements, which require various instruments and professional skills, thereby explaining the high costs and time required for the tests [4]. If the preprocessing step is not properly executed, dissolved red or white blood cells may be detected in plasma samples, compromising the entire measurement results. The separation of plasma from whole blood samples [5] or urine sample purification [6] is often required in medical diagnostics or analyses to eliminate any interference from blood cells or other impurities and enhance the sensitivity and reliability of assays. Conventionally, bulky and expensive centrifugation equipment is used in these processes, necessitating large samples and skilled technicians for operation [4]. These challenges limit the application of blood tests in resource-limited settings.

To enable LOC technology to meet the requirements of POC applications, LOC devices must facilitate sample preprocessing and provide rapid measurement readouts. Overall, the methods used to separate blood cells and plasma can be roughly divided into active and passive methods. Active methods primarily rely on mechanisms such as dielectrophoresis, acoustic waves, magnetic fields, or centrifugal forces [7,8,9,10]. These methods usually require the dilution of a blood sample before separation is performed. Additionally, these active forces make cell lysis relatively easy, causing cell debris to mix with plasma and potentially resulting in false signals. To generate a separation force, an external power supply is required, and this complicates the design and fabrication of LOC devices. By contrast, passive methods do not require an external power supply. They rely on mechanisms such as inertial forces [11], the Zweifach–Fung effect [12], and shear forces [13], and are typically used in filtration and LOC devices for the separation of cells from plasma. Despite the effectiveness of these passive methods, most of these methods exhibit complexities in their microfluidic design or require the predilution of blood samples. Filtration based on physical size constraints, including membrane- or column-based filtration [14,15,16,17,18,19], is a simple approach to on-chip plasma separation that is inexpensive and has seamless integration. Nevertheless, membrane- and column-based filtration methods have major drawbacks, including the leakage and hemolysis of red blood cells at high hematocrit levels due to clogging [14]. As an alternative, a sedimentation-based approach to plasma separation that depends on differences in the density of blood’s components has been developed [4,20,21,22]; this approach uses a simple microfluidic channel design and a self-contained system.

Most of the aforementioned plasma separation designs do not incorporate analyte measurement functionality. In the majority of LOC systems, fluorescence microscopy is the primary detection method for different assay techniques [22,23,24,25,26]. Label-free (LF) biosensors are typically used to determine the concentrations of biomolecules without the need for labels. These biosensors have several advantages over fluorescence detection and have attracted considerable attention. Over the past few decades, LF biosensors have been extensively used in biotechnology applications and medical diagnostics. As transduction devices, optical LF biosensors are widely utilized because of their high sensitivity, resistance to electromagnetic interference, compact design, simple optical readout, and multiplexing and remote-sensing capabilities [27]. Different optical designs and devices were used to determine the concentrations of analytes through measurands such as wavelength, intensity, and coupling angle and phase [28]. Surface plasmon resonance (SPR) and localized surface plasmon resonance biosensors are the most commonly used optical LF biosensors. However, to generate SPR, a metal layer is required, which leads to significant photon loss, resulting in a low Q-factor (Q = λ_0_/Δλ). In general, a lower Q corresponds to a broader Δλ, which limits the detection resolution [29]. Additionally, metal absorption inhibits transmission measurements, and the high metal loss also reduces the reflected intensity, thereby limiting the use of SPR in the reflection mode measurement. In contrast, guided-mode resonance (GMR) sensors, also known as resonant waveguide grating or photonic crystal slab sensors, are made of dielectric materials and can achieve narrow bandwidths (<1 nm). These sensors, which enable the measurement of small shifts in resonant wavelengths with high precision and at a high measurement resolution, have been extensively employed [30,31].

Many chronic diseases, such as chronic kidney disease, rely on detecting blood or urine biomarkers to screen and stage the disease or monitor the disease’s progression. In optical resonator biosensors, blood or urine samples should be purified to remove cells or impurities because these cells or impurities can interfere with the evanescent field, resulting in false signals. Despite the importance of this step, the majority of GMR microfluidic chips do not have a specific mechanism for separating cells from plasma. Instead, these chips often focus on the detection of biomolecules in buffer solutions [22,23,24,25] or use plasma or serum solutions spiked with biomolecules [32,33] as test models. To our knowledge, few studies have attempted to utilize membranes [34] or micropost arrays [19] to simultaneously separate cells from plasma and achieve LF detection [19,34]. In this study, we designed a new type of LOC system that includes an LF GMR sensor and a microfluidic chip with a sedimentation chamber. This design enables an optofluidic chip to simultaneously perform plasma separation and blood biomarker detection, which we believe can benefit POC applications.

## 2. Materials and Methods

**Figure 1:**
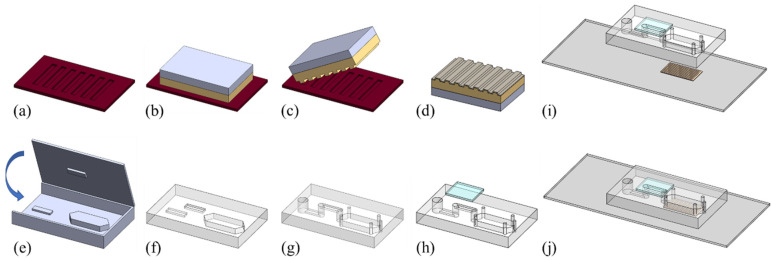
Fabrication of the (**a**–**d**) GMR biosensor and (**e**–**h**) microfluidic chip, and (**i**,**j**) the bonding process.

In this study, we developed an integrated optofluidic chip consisting of a GMR biosensor embedded in a microfluidic chip with a sedimentation filter for plasma separation. Figure 1 depicts the complete process used to fabricate the integrated optofluidic chip, including the fabrication of the GMR biosensor (Figure 1a–d) and microfluidic chip (Figure 1e–h) and the bonding process (Figure 1i,j).

### 2.1. Design and Fabrication of the GMR Filter

The GMR biosensor has a simple three-layer structure: a substrate layer with a surface-relief grating structure and a low refractive index (RI), a waveguide layer with a high RI, and a cover layer with target molecules and bulk solution. With appropriate device dimensions and material selection, this GMR filter can function as a bandstop filter given normal incidence [35,36]. Under broadband illumination conditions, a specific wavelength of light (the resonant wavelength) is reflected, with the remaining wavelength being transmitted. Experimentally, this phenomenon is observed as a narrow-band reflection or a transmission dip [37,38]. The resonant wavelength can be calculated using the following second-order Bragg condition [39]:

$$\lambda =n_{eff}\Lambda ,$$

where $n_{eff}$ is the effective RI of the structure and Λ is the grating period. In this case, $n_{eff}$ can be regarded as the weighted average of the RIs of the GMR structure [40]. Typically, the binding of the captured biomolecule changes the value of $n_{eff}$, which in turn changes the resonant wavelength. The degree of change in the resonant wavelength correlates with the number (or concentration) of adsorbed biomolecules.

**Figure 2:**
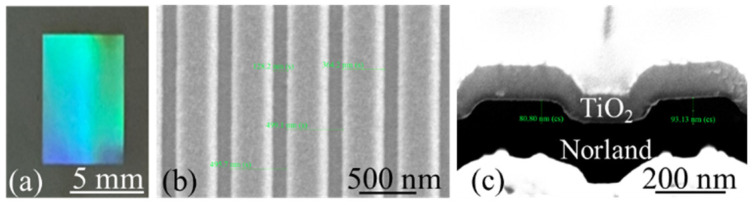
(**a**) GMR sensor. Scanning electron microscopy images of the GMR sensor: (**b**) top view and (**c**) cross-sectional view.

In this study, a GMR sensor was fabricated using an inexpensive replica molding technique. Briefly, electron beam lithography and reactive ion etching were used to pattern a grating pattern and transfer it onto a Si wafer (Figure 1a). Subsequently, this grating pattern was transferred to an ultraviolet-curable optical adhesive (NOA13825; Norland Products, Jamesburg, NJ, USA) by using a replica molding technique. Before replication, the Si master was immersed in Repel silane (Sigma-Aldrich, Burlington, MA, USA) for 2 min, after which it was rinsed with toluene, ethanol, and deionized (DI) water with sonication to prevent the stiction of NOA13825 to the Si master. Next, NOA13825 was sandwiched between the Si master and a polyethylene terephthalate (PET) sheet (Figure 1b). After the sample was cured through ultraviolet exposure, the NOA13825–PET combination was separated from the master (Figure 1c). Finally, a layer of TiO_2_ was sputtered onto the NOA13825–PET combination (Figure 1d). Figure 2a depicts the GMR sensor on a PET sheet with a grating area of 5 × 10 mm^2^. Scanning electron microscopy images of the GMR sensor revealed a grating period and depth of 496 and 93 nm from the top and cross-sectional views, respectively, and the TiO_2_ layer thickness was 97 nm (Figure 2b,c).

### 2.2. Design and Fabrication of the Microfluidic Sedimentation Filter

To achieve automatic on-chip blood plasma separation without hemolysis, a microfluidic channel with a sedimentation chamber was used, as described in the literature [41]. This sedimentation-based approach leverages gravity and density differences for the separation of blood plasma [4,22,41].

A fabrication process involving a polydimethylsiloxane (PDMS, SYLGARD 184 Silicone Elastomer Kit; Dow Corning, Midland, MI, USA) molding technique was used. Briefly, polymethyl methacrylate (PMMA) was used to fabricate top and bottom molds (Figure 1e). Subsequently, degassed liquid PDMS, with a base/curing agent ratio of 10:1, was gently poured inside the assembled molds. Once the PDMS–PMMA structure had been cured at 80 °C for 60 min in an oven, the PDMS was separated from the PMMA molds (Figure 1f). Next, vertical microfluidic channels were created using a biopsy punch to connect the top and bottom channels. Inlet, outlet, and ventilation holes were also created using a biopsy punch (Figure 1g). Finally, the top channel was sealed with a thin layer of PDMS (Figure 1h).

### 2.3. Integration of the Microfluidic Chip, GMR Sensor, and Detection Setup

To achieve simultaneous plasma separation and LF biomarker detection, the GMR sensor was integrated into the microfluidic chip. Briefly, the GMR sensor was attached to a glass slide by using NOA68 (Norland Products) with ultraviolet exposure (Figure 1i). Subsequently, uncured PDMS was used as an adhesive to irreversibly bind the PDMS microfluidic chip to the GMR sensor. A thoroughly mixed and degassed liquid PDMS mixture with a base/curing agent ratio of 10:1 was spin-coated on a glass slide at 3000 rpm for 60 s. After the microfluidic chip was gently pressed on top of the spin-coated PDMS to pick up a thin layer of uncured PDMS, it was placed on top of the GMR sensor. Finally, the integrated optofluidic chip was cured at 70 °C for 60 min in an oven.

### 2.4. Chip Preparation for the Detection of Blood Biomarkers

Human serum albumin is a vital biological macromolecule synthesized exclusively in the liver, with a normal serum concentration of 35–50 mg/mL [42]. Abnormal serum levels are associated with various diseases. For instance, elevated albumin levels in urine can signal early cardiovascular and kidney disease in diabetes and hypertension [42]. Additionally, abnormal serum concentrations in blood plasma may indicate cancer, rheumatoid arthritis, liver cirrhosis, liver failure, or chronic hepatitis [42,43]. Hence, in this study, albumin detection in blood samples was used as a test model to demonstrate the feasibility of the proposed plasma separation and biomarker detection approach in clinical settings. To achieve albumin detection, the surface of the GMR sensor was modified for antibody immobilization. Briefly, the GMR sensor–glass slide combination (Figure 1i) was treated with oxygen plasma to enrich the surface of TiO_2_ with hydroxyl groups. Subsequently, epoxy silane (1% [3-glycidoxypropyl]dimethoxysilane in toluene) was dispensed on the surface of the GMR sensor for 40 min at room temperature to form covalent bonds with the hydroxyl groups [44]. After the GMR sensor–glass slide combination was rinsed with acetone, ethanol, and DI water, it was blow-dried with N_2_ gas. Finally, the surface-modified GMR sensor was bonded with the microfluidic chip, as described in the previous section.

Anti-albumin antibodies were diluted in phosphate-buffered saline (PBS) to a concentration of 60 μg/mL, and the resultant solution was injected into the optofluidic chip. Subsequently, the optofluidic chip was maintained in a humid container for 8 h at room temperature to incubate the antibodies. After the antibody solution was aspirated out, PBS containing 0.05% Tween 20 (PBS-T) was injected into the optofluidic chip to remove unbound antibodies and wash the chip. Next, blocking solution (1× PBS 1% casein blocker; Bio-Rad Laboratories, Hercules, CA, USA) was injected and incubated for 1 h to passivate the GMR sensor and minimize subsequent nonspecific binding. After the chip was washed with PBS-T, fresh PBS was injected into the chip. Finally, transmission spectra were recorded every 10 s for 5 min as a baseline signal.

## 3. Results and Discussion

### 3.1. Filtration by the Microfluidic Channel with the Sedimentation Chamber

**Figure 3:**
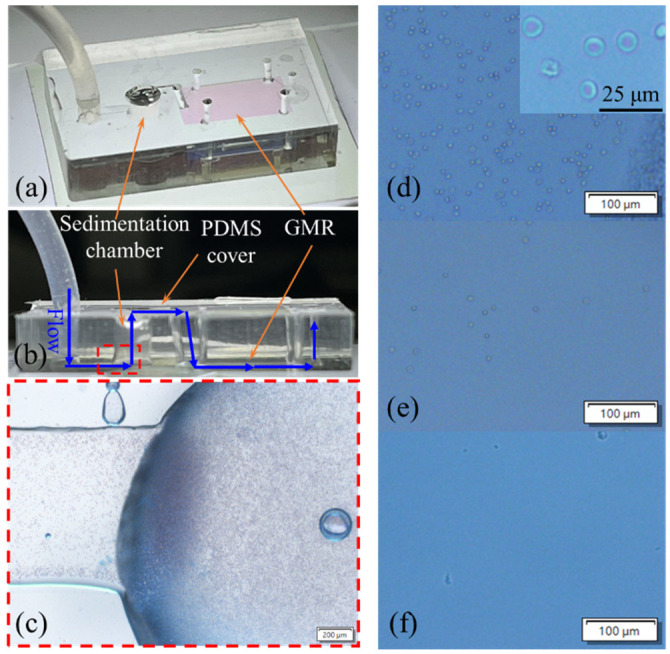
(**a**) Tilted and (**b**) side views of the optofluidic chip. (**c**) Top view of the microscopic image showing the entrance of the sedimentation chamber, highlighted by the red-dashed box in (**b**),with numerous blood cells. (**d**–**f**) Microscopic images of the GMR sensor region for flow rates of 5, 1, and 0.5 μL/min.

Figure 3a,b depict the tilted and side views of the optofluidic chip. The sedimentation chamber had a diameter of 1.5 mm and a height of 4 mm. After the sample was delivered through a tube, it flowed through the bottom channel and reached the sedimentation chamber. In this chamber, the cells were retained; only the plasma flowed to the top channel and reached the GMR detection region. This flow is indicated by blue arrows in Figure 3b. An automatic syringe pump (Fusion 100; Chemyx, Stafford, TX, USA) was used to inject the blood sample into the optofluidic chip at various flow rates. Finally, an inverted microscope (Olympus IX73; Olympus, Tokyo, Japan) was used to characterize the efficacy of blood plasma separation.

Diluted blood was injected into the optofluidic chip to test the separation of blood plasma. Before filtration, blood cells were observed in the sedimentation chamber (Figure 3c). Three flow rates (5, 1, and 0.5 μL/min) were initially tested. At flow rates of 5 and 1 μL/min, the separation process was unsuccessful because cells were observed in the GMR sensor region (Figure 3d,e). By contrast, no blood cells were observed when the flow rate was 0.5 μL/min. However, at this flow rate, blood was injected over a period of 60 min to fill the GMR sensor region. Therefore, to shorten the separation time, we adopted the two-stage loading approach proposed by Kuroda et al. [41]. Briefly, blood was injected at a flow rate of 5 μL/min to fill 80% of the sedimentation chamber. The injection process was then stopped for 10 min to enable the blood cells to sediment. The remaining blood was subsequently injected at a flow rate of 0.5 μL/min. With this approach, the total duration required to obtain plasma was 30 min. The separation time can be further reduced by reducing the optofluidic chip size, including the channel size and the GMR detection region.

### 3.2. Bulk RI Sensitivity Measurement

A simple transmission setup was used to monitor the resonant wavelength of the GMR sensor, with sucrose flowing through the GMR sensor region to characterize the sensor’s bulk sensitivity. A broadband light source was coupled to a fiber with a core diameter of 400 μm and a collimator at the exit. Subsequently, light was transverse-magnetically polarized before it hit the optofluidic chip, where it interacted with the GMR sensor. Finally, the transmitted light was collected by another fiber with a core diameter of 50 μm, which was connected to a spectrometer (USB2000+VIS-NIR-ES; Ocean Optics, Orlando, FL, USA) for spectrum recording.

**Figure 4:**
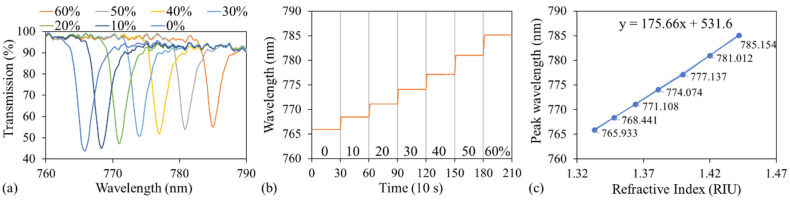
(**a**) Transmission spectra at different concentrations of sucrose. (**b**) Resonant wavelength as a function of sucrose concentration. (**c**) Resonant wavelength as a function of RI.

Sucrose solutions with concentrations of 0% (DI water), 10%, 20%, 30%, 40%, 50%, and 60%—with corresponding RIs of 1.333, 1.348, 1.364, 1.381, 1.400, 1.42, and 1.442 [45]—were used to characterize the bulk sensitivity and limit of detection (LOD) of the GMR biosensor. Before sucrose of another concentration was injected into the microfluidic channel, the previous solution was aspirated, and the channel was rinsed with DI water. As previously mentioned, light of a specific wavelength resonated with the GMR sensor, resulting in a dip in transmission at this wavelength. Figure 4a depicts the transmission spectra obtained for the various concentrations of sucrose. As the sucrose concentration increases, the corresponding RI also increases, leading to an increase in the $n_{eff}$ of the structure. Consequently, according to the second-order Bragg condition, the resonant wavelength (or dip wavelength) will also shift to a longer wavelength. At each concentration, transmission spectra were recorded every 10 s for 5 min (Figure 4b). The average dip (or resonant) wavelength was used to represent the resonant wavelength of each sucrose concentration (Figure 4c). The full width at half maximum (FWHM) slightly decreased with increasing sucrose concentration, decreasing from 2.51 to 1.93 nm as the concentration was increased from 0% to 60% (Figure 4a). As the sample’s RI increases, the RI contrast between the sample and TiO_2_ decreases, resulting in the better confinement of the guided mode within the structure, and a reduction in radiative or coupling losses. The enhanced field confinement leads to a sharper resonance with a narrower FWHM. The bulk RI sensitivity was defined as the ratio of the change in resonant wavelength to the change in RI, calculated as the slope of the linear fitted line minus 175.66 nm/RIU. The figure of merit (FoM), defined as sensitivity divided by the FWHM, was calculated as 69.98 RIU^−1^ when the FWHM at 0% was used. In addition, the bulk LOD, defined as three times the average standard deviation of all measured concentrations divided by the sensitivity, was calculated to be 2.21 × 10^−4^ RIU.

### 3.3. Recombinant Protein Detection

**Figure 5:**
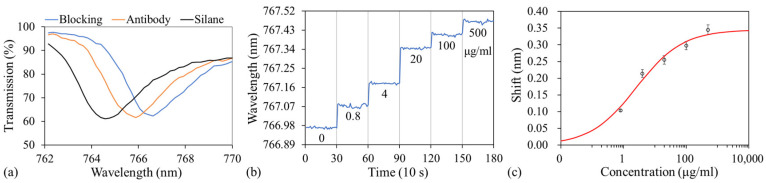
(**a**) Transmission spectra after silanization, antibody immobilization, and blocking. (**b**) Resonant wavelength as a function of antigen concentration. (**c**) Dose–response curve for albumin detection.

The transmission spectra were measured after silanization, antibody immobilization, and blocking. Figure 5a depicts the successful attachment of antibodies and subsequent blocking process; shifts of 1.3 and 0.74 nm, respectively, were measured in the resonant wavelength.

After casein was used for blocking, fresh PBS was injected into the optofluidic chip, and transmission spectra were recorded, with the resonant wavelength used as the baseline signal. Subsequently, the PBS was aspirated, and the lowest concentration of albumin (4 μg/mL in 1× PBS 1% casein blocker) was injected, after which it was left to incubate for 20 min. After the albumin solution was aspirated, the channel was rinsed with PBS-T. Finally, fresh PBS was loaded, and transmission spectra were obtained every 10 s for 5 min. The same procedure was performed for other concentrations of albumin (20, 100, and 500 μg/mL) for the same sensor. Figure 5b depicts the resonant wavelengths corresponding to different concentrations of albumin in a single set of experiments. The entire procedure was performed on another two optofluidic chips. A four-parameter logistic model was used to fit the experimental data in OriginPro 2016 (OriginLab, Northampton, MA, USA). Figure 5c shows the fitted dose–response curve for albumin detection. The value of the LOD, defined as the concentration corresponding to the shift associated with the blank sample (0%) plus three times the average standard deviation of the shift associated with all concentrations over the three experimental runs, was calculated as 0.158 μg/mL. The detection range and LOD achieved in this work are comparable to those reported in previous optical biosensors for detecting albumin in buffer solution [43,46,47,48].

Currently, for each run, different albumin concentrations were tested on the same sensor. Although a washing step was carried out between concentrations, only nominal concentrations can be clear. In the future, separate sensors can be used for each concentration, and experiments can be performed in triplicate to obtain a more reliable dose–response curve. Additionally, selectivity tests can be conducted to assess the nonspecific binding and evaluate the effectiveness of surface functionality. Furthermore, for simplicity, we employed a commonly used epoxy silane functionalization method, which enables covalent bonding with antibodies’ amino groups, resulting in random antibody orientation. This randomness may lead to reduced antigen-binding efficiency and, consequently, lower sensitivity. Future efforts to optimize antibody orientation, density, and binding efficiency will further enhance sensing performance.

### 3.4. Verification of the Sedimentation Chamber’s Efficacy

Healthy individuals have albumin levels ranging between 35,000 and 55,000 μg/mL. In this study, given the detection range identified from the standard curve (Figure 5c), blood diluted 1000 times in PBS was used to demonstrate the efficacy of the proposed optofluidic chip in blood plasma separation and albumin detection. An unfit blood sample was obtained from the Blood Center in Hsinchu, Taiwan, and three experiments were conducted to confirm the efficacy of the proposed optofluidic chip in simultaneous plasma separation and biomarker detection.

#### 3.4.1. Plasma Sample

Upper plasma (~85 μL) was extracted from the diluted blood sample and loaded into the antibody-immobilized optofluidic chip through simple erythrocyte sedimentation. After 20 min of incubation, the optofluidic chip was rinsed with PBS-T and PBS. Finally, the channel was loaded with fresh PBS. Overall, the results indicated a shift in the resonant wavelength of 0.292 nm. In accordance with the four-parameter logistic model outlined in Figure 5c, the concentration of albumin was thus estimated to be approximately 46.96 μg/mL.

#### 3.4.2. Blood Sample Processing with the Sedimentation Chamber

As previously described, the diluted blood sample was injected into the optofluidic chip in a two-stage loading procedure. Briefly, the sample was loaded into the channel at a rate of 5 μL/min to fill approximately 80% of the sedimentation chamber. The sample was then retained for 10 min to sediment blood cells. Blood was subsequently injected again at a rate of 0.5 μL/min until the plasma sample covered the GMR detection region. After 20 min of incubation, PBS-T and PBS were used sequentially to rinse the channel. Finally, fresh PBS was injected, and the resonant wavelength was measured. The results indicated a shift in the resonant wavelength of 0.289 nm. In accordance with the four-parameter logistic model, the concentration of albumin was estimated to be approximately 40.68 μg/mL. This concentration was close to that directly measured from the plasma sample, indicating the effectiveness of the sedimentation chamber.

#### 3.4.3. Blood Sample Processing Without the Sedimentation Chamber

**Figure 6:**
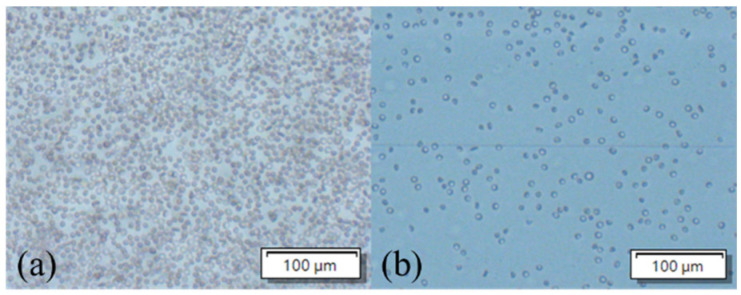
Microscopic image of the GMR sensor region (**a**) before and (**b**) after washing.

In this experiment, the diluted blood sample was injected into the GMR detection region from the outlet—it did not pass through the sedimentation chamber (Figure 3a, right). After 20 min of incubation, the same washing procedure was performed as described in the preceding section. The results indicated a shift in the resonant wavelength of approximately 3.26 nm, which was considerably larger than that observed at the highest concentration of 500 μg/mL (Figure 5c). Figure 6a shows a microscopic image of the GMR sensor region after the injection of the blood sample. Figure 6b depicts the blood cells remaining in the GMR detection region despite the washing procedure, indicating the importance of the sedimentation chamber for cell filtration.

## 4. Conclusions

In this study, we developed an integrated optofluidic chip by incorporating a GMR sensor into a microfluidic chip. This microfluidic chip includes a sedimentation chamber to enable the retention of blood cells and allow only plasma to flow into the GMR sensor for blood biomolecule detection. Our results indicated that the GMR sensor achieved bulk sensitivity and an LOD of 175.66 nm/RIU and 2.21 × 10^−4^ RIU, respectively. Further research can be investigated to optimize our GMR sensor design, including its dimensions, to achieve a higher sensitivity and LOD. The optofluidic chip successfully detected albumin in buffer solution with an LOD of 0.158 μg/mL within the concentration range of 0–500 μg/mL. Currently, for each run, different albumin concentrations were tested on the same sensor. Although a washing step was carried out between concentrations, only nominal concentrations can be clear. In the future, separate sensors can be used for each concentration, and experiments can be performed in triplicate to obtain a more reliable dose–response curve. In the future, selectivity tests can be conducted to assess the nonspecific binding and evaluate the effectiveness of surface functionality.

By following a two-stage injection approach (injection rate of 5 μL/min followed by 0.5 μL/min), we successfully separated plasma within only 30 min. Further optimization of the injection velocity and characteristics of the microfluidic chip—including its fluidic channel, sedimentation chamber, detection region, and flow rate—can shorten the overall separation time. Overall, we observed almost the same amount of shift in the resonant wavelength for the blood and plasma samples, indicating the effectiveness of the sedimentation chamber in separating blood cells from plasma, allowing only plasma to reach the GMR sensor for accurate detection.

In conclusion, our conceptual and practical model confirms the feasibility of our integrated optofluidic chip for the quantitation of biomarkers directly from blood samples. Future studies on a detailed investigation of batch-to-batch reproducibility in sensing performance will be critical for clinical applications. Additionally, a pump is currently required to deliver the sample. Integrating a manual pump design into the optofluidic chip could enable a fully automated operation, making it more suitable for practical POC applications. 

## Data Availability

Data is available on reasonable request from the corresponding author.
